# Amide proton transfer imaging has added value for predicting extraprostatic extension in prostate cancer patients

**DOI:** 10.3389/fonc.2024.1327046

**Published:** 2024-02-29

**Authors:** Xiaoyan Qin, Jian Lv, Jianmei Zhang, Ronghua Mu, Wei Zheng, Fuzhen Liu, Bingqin Huang, Xin Li, Peng Yang, Kan Deng, Xiqi Zhu

**Affiliations:** ^1^ Department of Radiology, Nanxishan Hospital of Guangxi Zhuang Autonomous Region, Guilin, China; ^2^ Department of Radiology, Graduate School of Guilin Medical University, Guilin, China; ^3^ Philips (China) Investment Co., Ltd., Guangzhou Branch, Guangzhou, China; ^4^ Department of Radiology, The Affiliated Hospital of Youjiang Medical University for Nationalities, Baise, China

**Keywords:** prostate cancer, extraprostatic extension, length of capsular contact, diffusion weight imaging, amide proton transfer, tumor size

## Abstract

**Background:**

Prostate cancer invades the capsule is a key factor in selecting appropriate treatment methods. Accurate preoperative prediction of extraprostatic extension (EPE) can help achieve precise selection of treatment plans.

**Purpose:**

The aim of this study is to verify the diagnostic efficacy of tumor size, length of capsular contact (LCC), apparent diffusion coefficient (ADC), and Amide proton transfer (APT) value in predicting EPE. Additionally, the study aims to investigate the potential additional value of APT for predicting EPE.

**Method:**

This study include 47 tumor organ confined patients (age, 64.16 ± 9.18) and 50 EPE patients (age, 61.51 ± 8.82). The difference of tumor size, LCC, ADC and APT value between groups were compared. Binary logistic regression was used to screen the EPE predictors. The receiver operator characteristic curve analysis was performed to assess the diagnostic performance of variables for predicting EPE. The diagnostic efficacy of combined models (model I: ADC+LCC+tumor size; model II: APT+LCC+tumor size; and model III: APT +ADC+LCC+tumor size) were also analyzed.

**Results:**

APT, ADC, tumor size and the LCC were independent predictors of EPE. The area under the curve (AUC) of APT, ADC, tumor size and the LCC were 0.752, 0.665, 0.700 and 0.756, respectively. The AUC of model I, model II, and model III were 0.803, 0.845 and 0.869, respectively. The cutoff value of APT, ADC, tumor size and the LCC were 3.65%, 0.97×10−3mm2/s, 17.30mm and 10.78mm, respectively. The sensitivity/specificity of APT, ADC, tumor size and the LCC were 76%/89.4.0%, 80%/59.6%, 54%/78.9%, 72%/66%, respectively. The sensitivity/specificity of model I, Model II and Model III were 74%/72.3%, 82%/72.5% and 84%/80.9%, respectively.

**Data conclusion:**

Amide proton transfer imaging has added value for predicting EPE. The combination model of APT balanced the sensitivity and specificity.

## Introduction

Prostate cancer (PCa) is the most common malignant tumor in men (1). Extraprostatic extension (EPE) is a critical pathological feature of PCa, and it poses a challenge for PCa treatment. The patients with EPE have higher positive margin rates and are prone to biochemical recurrence (2). Therefore, preoperative diagnosis of EPE is a vital factor, which directly affects the treatment and prognosis of patients (2). Extensive removal of positive margins can effectively reduce tumor recurrence if the tumor invades the capsule. However, expanding surgical scope can lead to impaired patient function for early-stage PCa lesions confined within the capsule. Achieving an optimal balance between the optimal surgical resection range and preserving bilateral neurovascular bundles to protect patient sexual function is a persistent challenge (3). Accurate preoperative prediction of EPE can help formulate surgical plans and achieve precise selection of treatment plans.

Multi-parametric magnetic resonance imaging (mp-MRI) is the most favorable imaging technique for local staging of PCa (4). At present, MRI examination is considered as the primary tool for preoperative prediction of EPE. The diagnosis of EPE primarily depends on the morphological indicators detected through the T2WI sequence. This sequence identifies the relationship between the tumor in the peripheral zone and the capsule, however, its sensitivity is weak and false negative rate is high (5). Currently, the potential of quantitative assessments of EPE with mp-MRI for improving accuracy and inter-reader agreement has been extensively studied (6). The Prostate Imaging-Reporting and Data System Version 2.1 (PIRADS V2.1) includes quantitative metrics such as the length of capsular contact (LCC), tumor size, and tumor volume to assist in predicting EPE (7). Previous studies have shown that these metrics improve the predictive value of mp-MRI for detecting EPE (8). However, in a previous study, it was reported that using apparent diffusion coefficient (ADC), LCC, and tumor size to predict EPE improved sensitivity but reduced specificity compared to subjective analysis, with no difference in overall accuracy (5). In a clinical setting, having a balance between sensitivity and specificity is crucial when determining appropriate treatment methods for detecting EPE.

A previous study has shown that the combination of amide proton transfer (APT) and ADC techniques complementarily improve the sensitivity and specificity in identifying PCa differentiation (9). APT imaging provides specific molecular information, which has added value in the diagnosis and risk stratification of PCa (9). APT is a novel magnetic resonance molecular imaging technique that is based on mobile proteins and peptides in cells, specifically used to reflect the increased concentration of proteins and peptides produced by mitotic activity and cell metabolism caused by abnormal protein synthesis in highly differentiated tumor cells (10). While tumor size and LCC reflect the morphological information of PCa, ADC and APT imaging techniques reflect differentiation and cell proliferation information of the PCa tissue. Given that these parameters reflect distinct information related to prostate cancer, creating a combined model using these parameters would be desirable to improve the assessment of EPE. Therefore, the objective of this study is to verify the diagnostic efficiency of tumor size, LCC, ADC, APT, and their combined models for predicting EPE.

## Materials and methods

### Subjects

**Figure 1:**
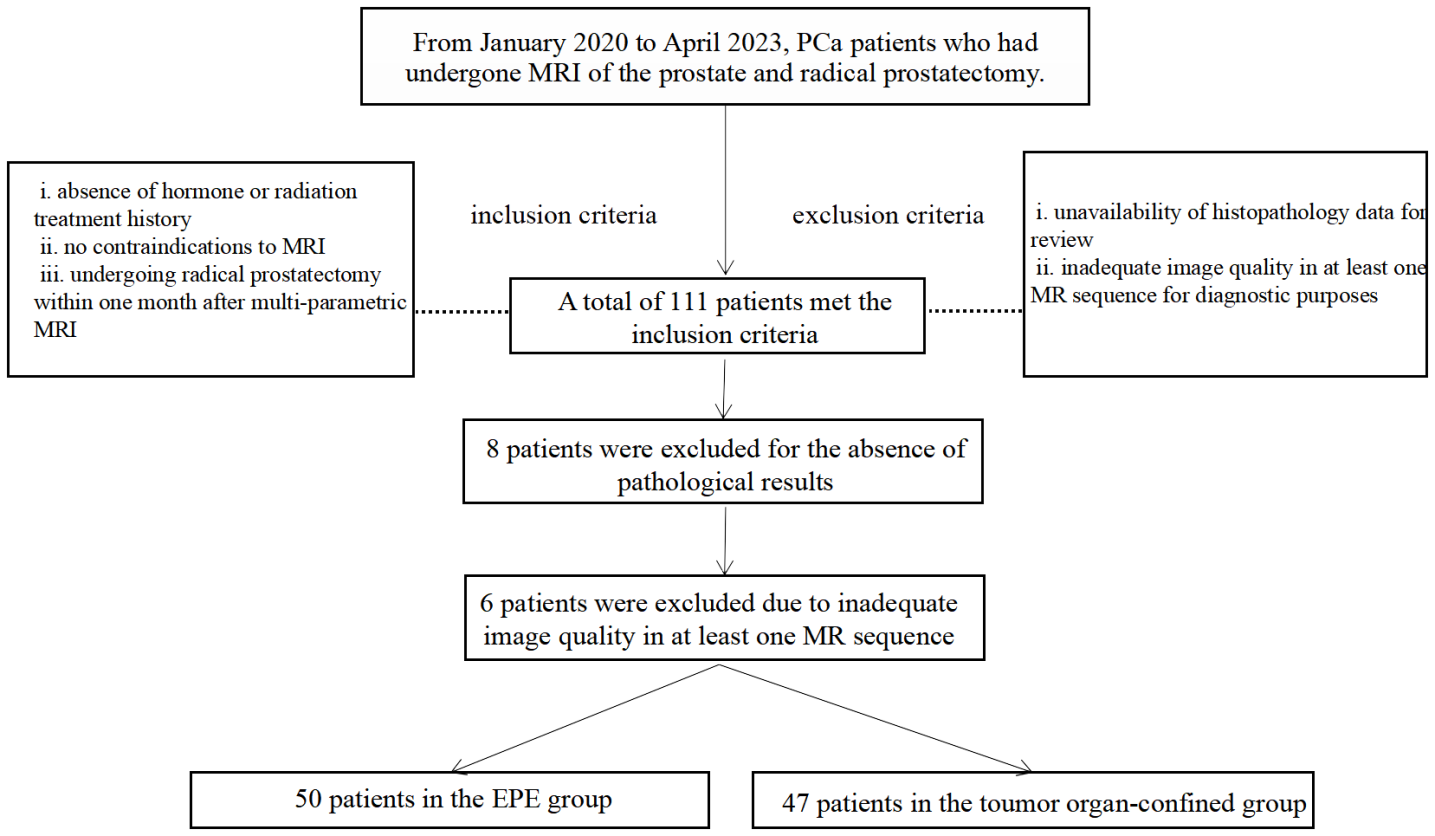
Flowchart of study participant selection. MRI, magnetic resonance imaging; PCa, prostate cancer; EPE, Extraprostatic extension.

This study is part of an ongoing investigation of PCa using multi-parametric MRI (retrospective analysis of prospectively-acquired data). The Institutional Ethics Committee of our hospital approved the study, and all subjects signed an informed consent form. We enrolled consecutively, from January 2020 to April 2023, patients with pathologically confirmed PCa who had undergone multi-parametric MRI of the prostate and radical prostatectomy at our hospital. The data were acquired based on the following criteria: inclusion criteria consisted of i) absence of hormone or radiation treatment history, ii) no contraindications to MRI, and iii) undergoing radical prostatectomy within one month after multi-parametric MRI. Exclusion criteria encompassed i) unavailability of histopathology data for review, and ii) inadequate image quality in at least one MR sequence for diagnostic purposes. **Figure 1** shows the flow chart of the enrolled patients.

### Multi-parametric MRI

The scans were performed using a 3.0 T MRI system (Ingenia 3.0 CX; Philips Healthcare, Best, The Netherlands) with a 16-channel phased-array body coil. The specific scan sequences used can be found in **Table 1**. During DWI sequence scanning, b values of 0, 100, 400, 800, and 1400 mm/s2 were used, with automatic calculation of the ADC map. Four Regional Saturation Technique slabs were used when APT scanning (9). A 2-second APT pre-pulse with a saturation power level of B1, rms=2 μT was achieved for APT imaging by transmitting dual radiofrequency channels in an interleaved manner. Nine frequency offsets (4.3 ppm, repeated 3 times at 3.5 ppm, 2.7 ppm, -2.7 ppm, -3.5 ppm, -4.3 ppm, -1560 ppm) relative to the water frequency were acquired for APT Z-spectrum. For the 3.5 ppm acquisition, a Dixon-based method was employed, and the acquisition window was shifted by ±0.4 ms and 0 ms, respectively. A B0 map was calculated from these three images and used for Z-spectrum correction.

**Table 1:** Sequences of multi-parametric MRI.

Scan sequences	Imaging plane	TR/TE, msec	FOV, mm2	Slice thickness, mm	Number of slices	Matrix size	Acquisition time,min
T1 TSE	Axial	450/10	200×200	3	24	308×264	1.34
T2 TSE	Axial/Coronal	2218/100	200×200	3	28	364×304	2.20
APT TSE	Axial	5000/8.3	100×100	8	10	64×45	7.00
DWI EPI	Axial	3826/69	200×200	3	28	152×125	4.47

APT, amide proton transfer; DWI, diffusion weighted imaging; FOV, field of view; MRI, magnetic resonance imaging; TE, echo time; TR, repetition time.

The APT(%) calculation method was as follows:

APT(%) = MTRasym (3.5 ppm)(%) = 100%∗ (S − 3.5 ppm−S + 3.5 ppm)÷ S0.

Note: MTRasym (3.5 ppm) is the abbreviation of magnetization transfer ratio asymmetry at 3.5 ppm. S0 represent the signal intensities without saturation pulse.

### Image analysis

**Figure 2:**
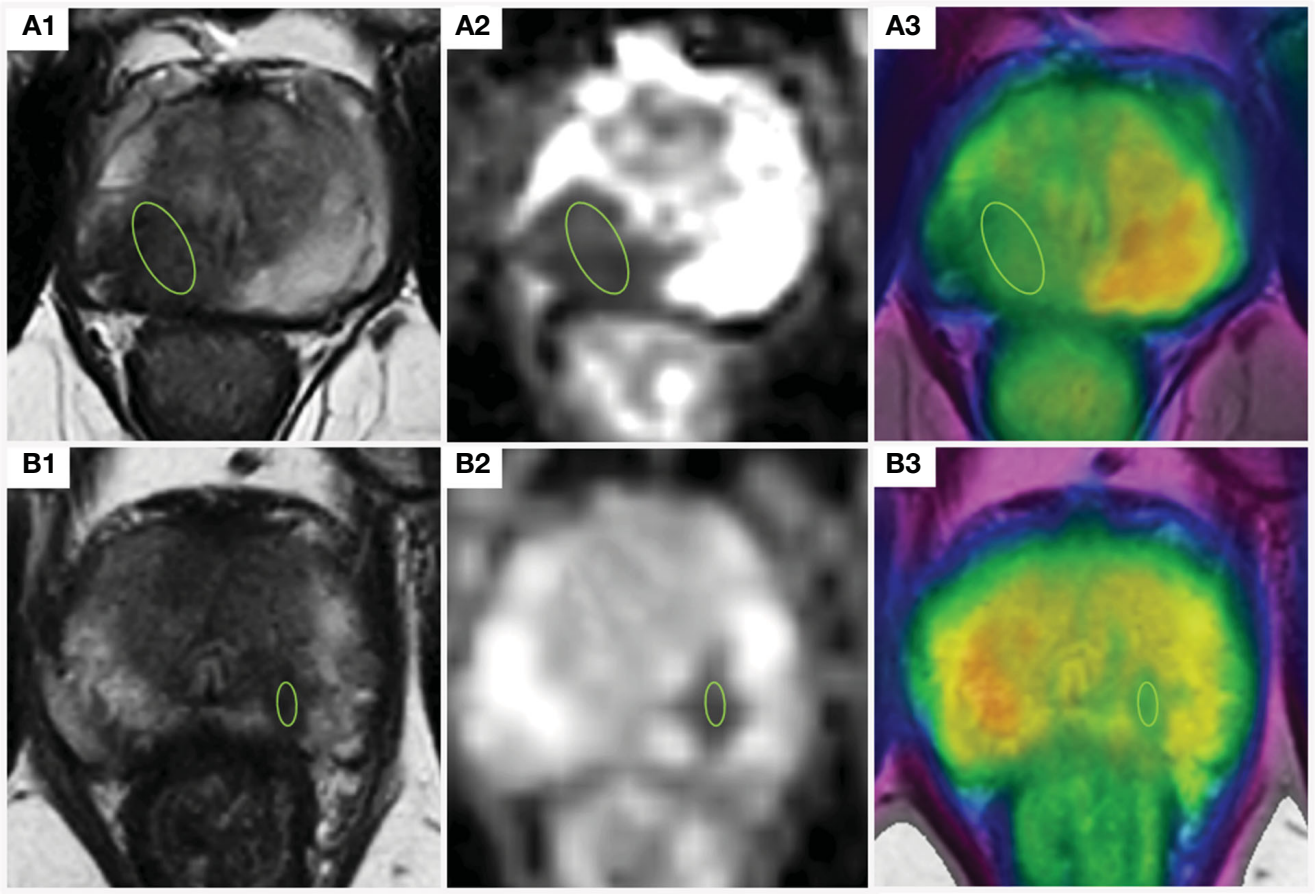
Indications of the definition of the ROIs for parameter analyses. **(A)** prostate cancer with EPE; A1,2: The lesion appeared hypointense on the T2-weighted image and the ADC map, ADC =0.92×10^−3^ mm^2^/s; A3: APT-weighted image with a T2WI overlay (APT =3.76%); **(B)** prostate cancer confined within the organ: B1,2: The lesion appeared hypointense on the T2-weighted image and the ADC map, ADC =1.26×10^−3^mm^2^/s; A3: APT-weighted image with a T2WI overlay (APT =3.32%); ADC, apparent diffusion coefficient; APT, amide proton transfer; TEPE, Extraprostatic extension; ROI, region of interest. T2WI, T2-weighted imaging.

The review of each examination was conducted retrospectively on the post-processing workstation of version 8 of “IntelliSpace Portal” (Philips Healthcare, The Netherlands). Two experienced radiologists (Zhu X and Qin X with 21 and 15 years of abdominal radiology experience, respectively) reviewed all MR images in consensus, without knowledge of the final histopathology results. In cases where multiple PCa lesions were present, the dominant lesion was selected for analysis. The dominant lesion was defined as a mass-like region with decreased T2 signal and ADC. For each dominant lesion, a region of interest (ROI) was set in three consecutive layers, maintaining a distance from the lesion’s edge to avoid volume effect (as illustrated in **Figure 2**). We used the “IntelliSpace Portal” workstation to conduct the image processing. In the IntelliSpace Portal, fusion approach was performed to draw the ROI. The APT and ADC values were calculated as the average values within the lesion ROI in different layers.

### The length of the capsular contact and tumor size

The radiologists evaluated each tumor foci for EPE utilizing a Likert scale that was previously described to increase the probability for EPE (5). T2-weighted imaging (T2WI) was utilized to measure the length of contact of each dominant lesion with the overlying capsule, using the curved measurement tool within our Picture Archiving and Communication System. During the independent review by both radiologists, the maximum transverse dimension of each tumor was measured on axial T2W-MRI.

### Pathologic assessment

The prostate specimens obtained from radical prostatectomy of each patient underwent formalin fixation, followed by standard step-sectioning with preparation of hematoxylin and eosin slides. The largest single area of tumor within the radical prostatectomy specimen was identified and mapped onto the gross digital images to enable accurate localization of tumors for quantitative measurements using MRI. This focus is referred to as the “dominant tumor focus” (11). In the current study, EPE was defined as the presence of any type of extracapsular extension. Organ confined disease was defined as the absence of these three conditions. The presence of EPE was recorded as 1, otherwise was recorded as 0. According the presence of EPE, individuals were divided into EPE group and tumor organ confined group.

### Statistical analysis

The normal distribution of the data was tested by Kolmogorove Smirnov method. After testing for normality, nominal data are presented as mean with standard deviation (SD). MRI parameters were compared between two groups using the independent sample t-test. Binary Logistic regression was used to screen predictors of EPE. Factors with P<0.05 were used as the input variables for the receiver operator characteristic (ROC) curve analysis. First, the ROC analysis was performed to assess the diagnostic performance of each variable for predicting EPE. Second, 3 combined model were established: model I (ADC + LCC + tumor size), model II (APT + LCC + tumor size), and model III (APT + ADC + LCC + tumor size) were established. Finally, the Youden index was calculated according to the following equation: Youden index = sensitivity + specificity –1. The cutoff value, sensitivity and specificity was selected based on the maximum value of the Youden index. P value<0.05 was considered a statistically significant result. All data were analyzed at the two-sided 5% significance level using SPSS 21.0.0 (IBM Corp., Armonk, NY, USA).

## Results

### Demographics characteristics and MRI derived parameters

Patient demographic and MRI-derived parameters are detailed in **Table 2**. The inclusion criteria were met by a total of 111 patients in this study. Of these, 6 patients were excluded due to inadequate image quality in at least one MR sequence for diagnostic purposes, and 8 patients were excluded for the absence of pathological results. Ultimately, 97 PCa patients, consisting of 50 patients in the EPE group and 47 patients in the organ-confined group, were selected for analysis. The age distribution was similar between the two groups, whereas significant differences in LCC, tumor size, APT, and ADC values were observed (*p<0.001*).

**Table 2:** Patient characteristics and comparison of parameters between two groups.

Parameters	Groups		t-value	*P*-value
	EPE (n=50)	Tumor Organ confined (n=47)		
Age (y), mean ± SD	61.51 ± 8.82	64.16 ± 9.18	1.448	0.151
APTw (%)	3.57 ± 0.0.57	3.18 ± 0.45	3.73	***<0.001***
ADC(×10−3mm2/s)	0.97 ± 0.35	1.19 ± .420	2.68	***0.008***
Tumor size, mm	17.06 ± 3.57	14.50 ± 3.20	3.78	***<0.001***
LCC, mm	13.38 ± 3.45	10.05 ± 3.07	4.91	***<0.001***

APTw, amide proton transfer-weighted; ADC, apparent diffusion coefficient; LCC, length of capsular contact; SD, standard deviation; mm, millimeter.

Bold font represents statistical significance.

### Binary logistic analysis of the association of metrics with EPE

Binary logistic analysis results were showed in **Table 3**. APT, ADC, tumor size and the LCC were all independent predictors of EPE. Odds ratio (OR) of APT, ADC, tumor size and the LCC were 4.362(1.700-11.194), 0.235(0.065-0.841), 1.264 (1.087-1.470), 1.398 (1.186-1.674), respectively, P value<0.05 for above mentioned parameters.

**Table 3:** Association of radiologic parameters with EPE.

Parameters	OR (95% CI)	*P* values
APTw (%)	4.362(1.700-11.194)	**0.002**
ADC(×10−3mm2/s)	0.235(0.065-0.841)	**0.026**
Tumor size, mm	1.264 (1.087-1.470)	**0.002**
LCC, mm	1.398 (1.186-1.674)	***<0.001***

APTw, amide proton transfer-weighted; ADC, apparent diffusion coefficient; LCC, length of capsular contact; EPE, Extraprostatic extension; Bold font represents statistical significance.

### ROC analysis

**Figure 3:**
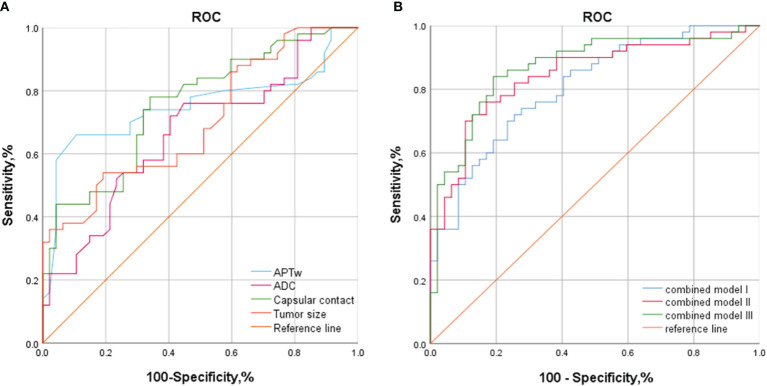
ROC analyses for assessing the diagnostic efficacy of MR parameters for predicting EPE. **(A)** ROC analyses for assessing the diagnostic efficacy of MR parameters for predicting EPE. **(B)** ROC analyses for assessing the combined models for predicting EPE. APT, amide proton transfer; ADC, apparent diffusion coefficient; LCC, length of capsular contact; EPE, Extraprostatic extension; Combined Model I, LCC + tumor size + ADC; Combined Model II, LCC + tumor size + APT; Combined Model III, LCC + tumor size + ADC + APT. Bold font represents statistical significance.

ROC analyses for assessing the diagnostic efficacy of MRI derived parameters were summarized in **Figure 3**, **Table 4**.

**Table 4:** Diagnostic Performance of variables for predicting EPE.

parameters	Cut off value	AUC	95% CI	Sensitivity	Specificity	P values
APT	3.650	0.752	0.649-0.855	76%	89.4%	***<0.001***
ADC	0.97	0.665	0.557-0.773	80%	59.6%	***0.005***
Tumor size	17.30	0.700	0.597-0.803	54%	78.9%	***0.001***
LCC	10.78	0.756	0.661-0.851	72%	66%	***<0.001***
Combined Model I	/	0.803	0.718-0.888	74%	72.3%	***<0.001***
Combined Model II	/	0.845	0.766-0.924	82%	75.5%	***<0.001***
Combined Model III	/	0.869	0.793-0.940	84%	80.9%	***<0.001***

APT, amide proton transfer; ADC, apparent diffusion coefficient; LCC, length of capsular contact; EPE, Extraprostatic extension; Combined Model I, LCC + tumor size + ADC; Combined Model II, LCC + tumor size + APT; Combined Model III, LCC + tumor size + ADC + APT.

Bold font represents statistical significance.

The area under the characteristic (AUC) curve values for APT, ADC, tumor size, and LCC were 0.752, 0.665, 0.700, and 0.756, respectively. The optimal cutoff values for APT, ADC, tumor size, and LCC were 3.65%, 0.97×10−3mm²/s, 17.30 mm, and 10.78 mm, respectively. The sensitivity and specificity values for APT, ADC, tumor size, and LCC were as follows: APT (76%/89.4%), ADC (80%/59.6%), tumor size (54%/78.9%), and LCC (72%/66%). (**Figure 3A**).

The AUC values for Model I, Model II, and Model III were 0.803, 0.845, and 0.869, respectively. The sensitivity and specificity values for Model I, Model II, and Model III were as follows: Model I (74%/72.3%), Model II (82%/72.5%), and Model III (84%/80.9%). (**Figure 3B**).

## Discussion

This study demonstrated that mp-MR parameters, including APT, ADC, tumor size, and LCC were significantly associated with EPE. Different parameters had varying levels of sensitivity and specificity. The diagnostic accuracy of the combined models of the aforementioned parameters outperformed that of any parameter alone. More importantly, the combination models balanced the sensitivity and specificity of those variables for predicting EPE.

The LCC has been reported to provide fair to good performances for predicting EPE (5, 12). The PIRADS V2.1 guidelines introduced the MR imaging finding of a tumor-capsule interface greater than 10mm, linking it to EPE (13). The LCC had a moderate diagnostic performance in detection of EPE has become a consensus of researchers. In the present study, we reported a AUC of 0.756 (95% CI:0.661-0.851) for predicting EPE, the sensitivity and specificity were 72% and 66%, retrospectively. Several previous studies were in consistent with our results. Washino et al. reported that the LCC (odds ratio 1.079, p = 0.001) were independently associated with EPE and the AUC for detecting EPE was 0.70 (14). Onay et al. reported that the LCC provides fair diagnostic performance (AUC: 0.73) and reveals moderate sensitivity (69%) and specificity (68%) for detecting EPE in PCa (15). In another study, Onay et al. found that at the most optimal threshold of 13.5 mm, the sensitivity and specificity in predicting EPE were 75% and 52%, respectively (16). A recent meta-analysis reported that summary sensitivity and specificity were 0.79 (95% CI: 0.73–0.83) and 0.67 (95% CI 0.60–0.74), respectively, and the summary ROC was 0.81 (95% CI: 0.77–0.84) (17). Another meta-analysis summarized that the pooled sensitivity and specificity were 0.79 (95% CI 0.75–0.83) and 0.77 (95% CI 0.73–0.80) using LCC for predicting EPE (18).

However, different studies also show the heterogeneity of the results. The first is the heterogeneity of the degree of association between LCC and EPE, the association of every 1 mm increase in the measurement of LCC with the increase in the risk of EPE range from 4% to 13% (19, 20). The second is the heterogeneity of sensitivity and specificity. A recent meta-analysis showed the sensitivity ranging from 59% to 91% and the specificity from 44% to 88% in those included articles (17). Finally, the optimal threshold is associated with the balance between the sensitivity and the specificity (5, 12, 15). The recent studies that evaluated LCC as an indicator for EPE established quite different median values and thresholds, (ranging from 6 mm to 20 mm), and there is currently no consensus on the best cutoff value for predicting EPE. Consequently, determining the optimal threshold has become an essential topic of debate (16). Valentin and colleagues highlighted that specific LCC cutoff values correspond to varying levels of sensitivity and specificity; for instance, increasing the LCC cutoff from 7.55 mm to 20.5 mm reduces sensitivity from 98.3% to 45% and boosts specificity from 42.1% to 88.2% (21).

Previous studies have demonstrated an association between tumor size and EPE, with our study finding a significant relationship between larger tumor size and increased EPE risk (OR: 1.264, 95% CI: 1.087-1.470, p<0.001). Our findings align with those of Lim and colleagues, who concluded that a simple measure of maximal transverse tumor size is a reliable means of diagnosing EPE (22). According to PI-RADS v.2 guidelines, the optimal threshold for predicting EPE is 15 mm; In addition, two other studies have found that a cutoff value between 16 mm and 18 mm provides the best diagnostic performance (23, 24). These studies were consistent with our results. In our study, the optimal cutoff value was 17.30 mm, which aligns with the size threshold proposed by two other studies (5, 22). In the present study, we reported a AUC of 0.756 (95% CI:0.661-0.851) for predicting EPE, the sensitivity and specificity were 54% and 74.9%, retrospectively. Schieda et al. indicated that tumor size of 16 mm resulted in an AUC value of 0.77 (95% CI: 0.58-0.95) for diagnosing EPE using tumor size (24). The optimal cutoff value supported by Li et al. for diagnosing EPE through tumor size was 15 mm, yielding sensitivity and specificity values of 67% and 70%, respectively (18). According to Li et al., based on meta-analysis of five studies, the summary sensitivity and specificity values for diagnosing EPE using tumor size were estimated to be 62% and 75%, respectively (18). Furthermore, some studies reported greater specificity than sensitivity in predicting EPE through tumor size. Lim et al. suggested that tumor size of 15 mm resulted in a sensitivity/specificity of 72.4%/64.9% for diagnosing EPE, and supported that objective evaluation through tumor size improved sensitivity of diagnosis compared to subjective assessment (22). In the study by Schieda et al., the sensitivity and specificity values of tumor size for predicting EPE were estimated as 69.2% and 66.7%, respectively (24).

Currently, the relationship between the LCC, tumor size and the amount of EPE cannot be comprehensively understood yet. Results variability in different articles may be due to factors such as tumor grading, MRI readers’ experience, and differences in the signal acquisition coil (17). Additionally, variability in results may also be caused by differences in data measurement methods and location. For example, using a curvilinear method to measure LCC may result in more accurate results than using linear measurements (17). Quantitative analysis provides several potential benefits, such as improving accuracy, interobserver agreement, and histopathology correlation when compared to subjective assessments that mainly depend on radiologists’ personal pattern and experience (24). Nonetheless, various measurement methods, tools, MRI techniques, and sequences can affect the final results and, consequently, lead to widely varied optimal cutoff values (6, 8). LCC, tumor size, and ADC exhibited moderate diagnostic performance in predicting EPE. Among these measurements, LCC presented greater accuracy. Nevertheless, establishing an optimal cutoff threshold for clinical application is required due to the wide variation in values (18). LCC and tumor size improved sensitivity but reduced specificity compared to subjective analysis, with no difference in overall accuracy (5, 22). Tumor size seems to be the least critical independent variable. However, whether tumor size is an independent predictor of prognosis after considering grade, stage, and margins remains controversial (24).

However, the dominance of grade over pathological stage is evident. A tumor with Gleason Score (GS) 6 and EPE has a relatively favorable prognosis compared to a GS 9-10 tumor confined within the organ. High-grade cancer often involves seminal vesicle invasion and lymph node metastasis (25). Consequently, quantitative parameters that reflect the pathophysiological features of PCa have the potential to improve the accuracy of predicting EPE. Previous studies demonstrated that ADC and APT can reflect tumor tissue atypia, tumor cell increment, and tumor grade (10, 26). Studies have revealed that as tumor grade increases, there is a corresponding trend of increasing cellular density, loss of normal glandular structures, and a decrease in the extracellular space. This limits water diffusivity and results in lower ADC values (26). Kim et al. have found mean ADC to be useful in diagnosing EPE (27). Granja et al. predicted EPE using ADC and obtained a sensitivity of 83% with a specificity of 61% at the cutoff value of 0.87×10−3 mm2/s (28). While Ito et al. reported a sensitivity of 84.2% and a specificity of 59.0% at the cutoff value of 0.63×10–3 mm2/s (29). The reported sensitivity and specificity in above mentioned studies were similar to our results. According to a meta-analysis, the pooled sensitivity was 80.5%, while specificity was 69.1% (30). This sensitivity is similar to our result, but our specificity for predicting EPE with ADC values was lower (59.6%). Krishna et al. argued that the largest cross-sectional diameter and tumor size, ADC values tend to have elevated sensitivity rather than specificity (5, 22). Ito et al. reported that the combination of LCC and ADC cutoff values yielded an area under the curve (AUC) of 0.82. Their specificity (84.6%) and accuracy (81.0%) of the combined values were superior to their individual values (29). In our current study, the combination of ADC with LCC and tumor size (Model I) yielded an AUC of 0.803. This combination balanced the heterogeneity of sensitivity and specificity. In addition, it has been reported that mean ADC values alone are not useful for assessing EPE (11). Lim et al. reported that ADC entropy improved EPE prediction sensitivity, but mean ADC values and ADC ratio of tumor were not associated with EPE (11). This discrepancy may be related to the grading of tumors in the patients included and the sample size. Including a more balanced distribution of sample sizes for different Gleason grades could have improved the results for diagnosing EPE with ADC values (11). In addition, the heterogeneity of PCa differentiation is another possible reason, where lower percentile ADC values reflect poorly differentiated tumor tissue more easily, thus reflecting the biological activity of PCa in different populations (31).

Similar to ADC, APT is also an MR imaging marker that can reflect information about tumor pathophysiology. APT imaging is specific in detecting not only cellular density but also the rate of tumor cells proliferation, which elevates the overall protein levels in the tumor (9). APT values had been approved to be a discriminator of PCa in previous studies (10, 32). There is evidence indicating that APT imaging accurately reflects PCa aggressiveness. APT imaging reflects the elevated protein and peptide concentrations because of abnormal tumor cell proteosynthesis, mitotic activity, and altered cell metabolism, particularly in high-grade tumors (33). According to Yin et al., APT imaging accurately diagnoses PCa and strongly correlates to the GS, which is crucial in assessing the risk associated with PCa (34). Jia et al. suggest that APT imaging is a reliable method of distinguishing between low and high-grade cancers and detecting the difference in cancer aggressiveness in PCa management. In differentiating benign from malignant tissue, ADC MRI might be preferable, while APT MRI could be used to evaluate tumor aggressiveness in patients with PCa (35, 36). As an example, the AUCs were 0.983 for ADC and 0.601 for APT in distinguishing malignant tumors and benign regions. For separating low-grade tumors from high-grade tumors, the AUCs were 0.912 for APT and 0.734 for ADC (32). Qin et al. Reported that the combination model of APT and ADC can improve the diagnostic efficacy in differentiating the grades of PCa (9). Hu et al. showed that the combination of APTw and intravoxel Incoherent Motion Imaging, could enhance diagnostic performance in predicting PCa metastasis (37). Our research proved this, demonstrating an AUC of 0.845 for LCC and tumor size combination, and when combined with APT (Model II), the AUC was 0.869 after further inclusion of ADC (Model III). More importantly, the combination model balanced the sensitivity (84%) and specificity (80.9%). According to Qin et al., APT had higher specificity and lower sensitivity in PCa grading than did ADC. Conversely, ADC had higher sensitivity and lower specificity. Therefore, the combination of APT and ADC can complement each other in PCa grading, achieving higher accuracy (9). Compared to the values of ADC or APT, the combination model achieved a better balance of sensitivity and specificity (9). The balance of sensitivity and specificity of the combined model may be related that ADC and APT reflect different pathophysiological mechanisms of prostate cancer. ADC is mainly influenced by water diffusion at the cellular level. APT imaging reflects increased concentrations of proteins and peptides in mitotic activity and cellular metabolism caused by abnormal protein synthesis in tumor cells, which is commonly altered in high-grade tumors. Theoretically, APT imaging may be more specific to detect not only cell density but also the rate of tumor cell proliferation leading to overall mobility rising protein levels.

On the clinical setting, the results of this study have significant clinical applications, as a high sensitivity or specificity reading might be useful in different clinical settings (17). Accurate preoperative prediction of EPE is important for the choice of clinical treatment options. Patients with prostate cancer can undergo nerve-sparing radical resection, while patients with EPE may require radical resection without nerve-sparing, or neoadjuvant therapy. High sensitivity is required when selecting optimal patients choosing candidates for nerve-sparing radical resection. On the other hand, high specificity could be favored when there is a need to guard against overtreatment (38). Consequently, we believe that based on our study’s findings, APT imaging and its combined model would provide additional value in accurately assessing EPE, particularly in clinical settings where there is a need for the balance of sensitivity and specificity. In this study, EPE was predicted by some imaging features such as tumor size, LCC, ADC, and APT. Measuring these features can improve the robustness of EPE predictions, as they have been shown to be independent predictors of EPE. But more studies are needed to standardize and further refine existing MRI protocols to enhance the detection of EPE and subsequent risk stratification. For example, some nomograms and scoring systems have been developed to predict EPE, but their accuracy varies (8). Using the current PI-RADS v2 MRI staging guidelines has high specificity but lacks sensitivity (8). APT and its combined model demonstrate potential value in predicting EPE, but its clinical utility needs to be further verified in subsequent studies.

There are still several potential limitations of our study. First, the present study used only a dichotomous scheme of presence or absence for EPE, and did not study the predictive efficacy of the parameters of the MRI sources for different grades of EPE. Second, this study did not include other variables such as PCa GS score and clinical stage that might affect the predictive efficacy of EPE. Third, The sample size in our study was relatively small as it was a single-center cross-sectional observational study. Before introducing APT to predict EPE in clinical practice, a longitudinal study with a larger sample size will be necessary in the future.

## Conclusion

APT, ADC tumor volume and LCC were identified as independent predictors for predicting EPE. APT imaging and its combined model could provide added value in predicting EPE. More importantly, the combination model balanced the sensitivity and specificity. These findings have important clinical implications in the selection of appropriate management strategies for clinically significant PCa.

## Data availability statement

The raw data supporting the conclusions of this article will be made available by the authors, without undue reservation.

## Ethics statement

The studies involving humans were approved by Ethics Committee of Nanxishan Hospital of Guangxi Zhuang Autonomous Region. The studies were conducted in accordance with the local legislation and institutional requirements. The participants provided their written informed consent to participate in this study.

## Author contributions

XQ: Writing – original draft, Writing – review & editing. JL: Writing – original draft. JZ: Writing – original draft, Data curation, Formal analysis, Methodology, Project administration. RM: Writing – original draft. WZ: Writing – original draft. FL: Writing – original draft. BH: Writing – original draft. XL: Writing – original draft, Data curation, Methodology. PY: Data curation, Formal analysis, Writing – original draft. KD: Data curation, Methodology, Writing – original draft. XZ: Data curation, Methodology, Conceptualization, Formal analysis, Funding acquisition, Investigation, Project administration, Resources, Supervision, Validation, Visualization, Writing – original draft, Writing – review & editing.
